# The Role of Identity and Ontological (In)Security in Return Migration: an Empirical Perspective from Hungary

**DOI:** 10.1007/s12134-022-00964-z

**Published:** 2022-05-13

**Authors:** Gabor Lados, Gabor Hegedus, Zoltan Kovacs

**Affiliations:** 1 Institute for Regional Studies, Centre for Economic and Regional Studies, Pecs, Hungary; 2 Department of Economic and Social Geography, University of Szeged, Szeged, Hungary; 3 Geographical Institute, Research Centre for Astronomy and Earth Sciences, Budapest, Hungary

**Keywords:** Return migration, Adaptation, Cultural identity, Ontological security, Hungary

## Abstract

East–West migration has become a dominant spatial phenomenon in Europe since the step-by-step enlargement of the EU, posing considerable socio-economic and demographic challenges for sending countries. However, little is known about the geopolitics and motivations that inspire a return to the country of origin. The objective of this article is to identify the motivations for emigration, adaptation experiences and decisions of Hungarian migrants who worked in the West for a period of time and returned to Hungary, based on their skills and family status before their return. The research is based on in-depth interviews with 48 returnees and builds on the cultural identity model and the notion of ontological security. The study demonstrates the growing role of geopolitics in return migration, although its relevance differs in various groups, with clear distinctions between the high- and low-skilled migrants. The role of family as a geopolitical unit is increasing; factors like their endeavour to hold the family together, fear of children’s assimilation, loss of identity and uncertainty while abroad are important in shaping decisions to return. On the other hand, schemes to enhance the return migration of the highly skilled also build on geopolitical and nationalistic rhetoric, which appear to target experiences of ontological (in)security among migrants.

## Introduction

The scale of international labour migration has been steadily increasing due to globalisation, the accelerating mobility of labour, widening core–periphery relations, ongoing socio-cultural transformations and the increasing embeddedness of people and firms into transnational networks (Conway & Potter, 2009; Massey, 1993). The countries of Central and Eastern Europe (CEE) were hermetically cut off from international labour migration by the Iron Curtain, the world’s most heavily fortified frontier imposed by Stalin’s Soviet Union after World War II. In the following four decades, CEE countries belonged to the geopolitical hinterland of Moscow. Travel to and from these countries was strictly controlled by governments, and the international mobility of labour was practically non-existent (Turnock, 2003). National labour markets relied predominantly on domestic workforces under systems of central planning, egalitarianism and homogenisation. The international exchange of labour was possible only under strict bilateral government agreements which played, by and large, a very limited role; one such was the agreement between Hungary and the German Democratic Republic in the second half of the 1960s.

After the fall of the Iron Curtain, through the dismantling of central planning and the shift to a market economy, the CEE countries started to reintegrate into the global economy. Loosening border controls and the liberalisation of labour markets resulted in accelerating international labour mobility (Fassmann et al., 2014). Post-communist transformation coincided with the growing demand for skilled labour in the core countries of the European Union (EU-15) in the 1990s. Higher salaries, better working conditions, higher levels of social security and other benefits resulted in accelerating East–West migration.

Growing emigration to Western European countries was enhanced by the accession of 11 CEE countries to the EU in 2004, 2007 and 2013, resulting in what is commonly known as a brain drain process (Glorius, 2018; Nadler et al., 2016; Smoliner et al., 2013; Zuk et al., 2019). As of 2019, approximately nine million people from CEE countries have moved to other EU member states (predominantly EU-15 countries). Regions that are the most severely affected by population loss caused by emigration within the EU are located nearly exclusively in post-communist CEE countries. As European economic integration deepened in the new millennium, CEE migrants—or at least their labour—were very much welcomed in core countries of the Union. The white, Christian and relatively skilled labour force appeared to be more palatable and desirable in West European public opinion than a potential workforce envisaged as uninvited asylum-seekers from Asia or Africa (Hyndman, 2012).

The main objective of this article is to explore the motivations for emigration, adaptation experiences and decisions of Hungarian migrants who worked in the West for a period of time and returned to Hungary. Our aim is to identify the main factors that stimulated the migration decisions of people with different work experiences, skills and family status both before they left the country, while they stayed in the host country and when they returned. The study builds on the concepts of the cultural identity model or CIM (Sussman, 2011) and ontological (in)security (OS) when exploring migrants’ motivations and decisions (Botterill et al., 2020). During the research, in-depth interviews were carried out with skilled and less-skilled individuals who returned to Hungary between 2012 and 2015 after a stay abroad of between 1 and 10 years.

In the next section, we introduce the theoretical background to the research, with special attention paid to CIM and OS. Then, our research methods are introduced, followed by a consideration of some of the important dimensions of East–West migration and return migration with special reference to Hungary. Our empirical research findings are then presented before, in the final section, we discuss them and draw some conclusions.

## Identity and Ontological (In)Security: the Case of Intra-EU (Return) Migration

Academic interest towards return migration has clearly grown in Europe since the gradual enlargements of the EU and the intensifying East–West migration (Apsite-Berina et al., 2018, 2020; Glaser & Habers, 1974; Konzett-Smoliner, 2016; Nadler et al., 2016; Sardinha & Cairns, 2017; Vathi & King, 2017). There is a wide and ongoing debate on the causes and effects of return migration, the relationships between returnees and the economic development of their home country, and the challenges caused by reintegration (Berry, 1997; Cassarino, 2004; de Haas, 2010; King & Christou, 2010; Kunuroglu et al., 2016; Sardinha & Cairns, 2017). Return migration may cause multiple effects at different scales, such as at the macro-geographical scale (nation, region) and at the micro-scale (family, individuals). The beneficial effects of the phenomenon—such as the labour market use of material, intellectual and relational capital obtained abroad, the contribution to modernisation in the form of technological and other innovations and confidence-building between members of society—are all widely acknowledged, at least in terms of their theoretical potential (Klein-Hitpaß, 2016; OECD, 2008). However, research findings also confirm the negative effects that return migration may have: for example, returnees may find it difficult to reintegrate into their homeland’s economy and society and their material prosperity and ‘success stories’ may give rise to envy and emigration among their fellow citizens. These experiences may generate re-emigration or circular movements of the skilled or less-skilled (Gmelch, 1980; OECD, 2008; van Houte & Davids, 2008).

In the context of intra-EU (return) migration, the literature has suggested a somewhat fluid status as a result of access to mobility and EU citizenship rights (Aksakal & Schmidt, 2020; Demay & Mercenier, 2016; Favell, 2016). It is still unclear to what extent the different forms and dimensions of inequality across the EU translate into barriers of integration and experiences of marginalisation among EU migrants, all of which influence migrant identities and trajectories, including their possible return to the country of origin or circulatory migration (Favell, 2016). The literature in this area is limited, even though the concepts of return migration and reintegration have generated increasing interest recently (Konzett-Smoliner, 2016; Kordel & Lutsch, 2018; Lados & Hegedűs, 2019; Nadler et al., 2016; Remennick, 2021; Smoliner et al., 2013). This paper proposes the adoption of two concepts that seem to be especially relevant to a better understanding of the relations of return migrants with their homeland and their possible reintegration at the personal level, as well as their motivations before and after return: the cultural identity model (CIM) and the concept of ontological security (OS).

Although a growing interest towards the identity shifts of return migrants can be witnessed in the literature (Gullahorn & Gullahorn, 1963; Sener, 2020; Sussman, 2011; Wickens et al., 2020), it has not yet become part of mainstream theories. Sussman’s (2011) CIM underlines the shifts in the personal attributes of migrants from a socio-psychological perspective. The model—based on the case of Hong Kong returnees—focuses on the attachment of migrants to the home vs host country and changes during the migration process (i.e. before and during emigration and after return), analysing migrants’ cultural adaptation in the host country while maintaining their home identity. This theoretical construct helps us to understand the identity changes of returnees and to hypothesise their possible future migration decisions.

According to Sussman, the adaptation of host cultural values takes place in different ways while abroad. She argues that four different identity types (and shifts) of returnee might occur, which express a relationship to the identity of the country from which the returnees originate. These are *affirmative*, *subtractive*, *additive* and *global identity*. When there is an *affirmative* identity shift, returnees are firmly attached to their home-culture identity which is reinforced after return; they feel much better in their home country and are not so adaptive towards the host country’s social values and culture. Their psychosocial well-being can be much better secured at home (Wright, 2009).

However, members of the *subtractive* and *additive* groups experience significant stress upon return; in both cases, there is an isolation from and limited interaction with the home country culture while abroad. *Subtractive* returnees are not intimately tied to the culture of either the home or the host country. During their emigration, they tend to avoid public events and feel comfortable with their compatriots. This is partly due to their limited language skills; hence, their integration into the host society is often unsuccessful. They also face difficulties during return (e.g. reintegration into the labour market) and, due to stress, they often become alienated from both their host and their home countries.

In many respects, *additive* returnees resemble the subtractive group; the main difference is the relationship to their home country culture. Additive returnees retain and practise their own culture and traditions but are much more open to new influences experienced in the host country and usually adopt certain elements (e.g. attitude, work ethics, lifestyle). After returning, despite the stress, their connections with the host country are retained. If changing their cultural identity after returning is difficult, then a re-emigration (a second emigration following return) of the additive group is very likely, as was demonstrated by Sardinha & Cairns (2017) in their case study of Portuguese return migrants.

Fourth, less-common group is comprised of individuals carrying multiple identities, who could be called *global-identity returnees*. They can follow and adapt individual cultural traits according to their current working and living conditions. However, this is facilitated neither by the mixing of cultural values from the home and the host country nor by the development of a bicultural strategy. Members of this group consider themselves as world citizens, so they can flexibly and quickly adapt to social requirements anywhere abroad. They are moderately positive about their return and ready to move abroad in the future for a shorter or longer period (Kunuroglu et al., 2016; Sener, 2020; Sussman, 2011).

Further empirical research has highlighted that migrants’ motivations to return, their reintegration and their overall well-being are contingent upon personal circumstances and contextual factors beyond the ‘home culture’ (Vathi & King, 2017). The concept of OS may offer a useful tool to explore how national identity, family histories and social relationships shape migrants’ (in)securities and their behaviour (Botterill et al., 2020) and ultimately their reintegration or re-migration. OS was pioneered in the psycho-analytical literature by Laing (1960) and later adopted and developed in social theory by Giddens (1991). According to the concept, in addition to physical security, people also seek ontological security or the security of the self. In this regard, ontological insecurity means a state of general anxiety which stems from the disruption of habits and the inability to sustain a coherent narrative about doing, acting and being (Kinnvall, 2004). According to Rumelili (2015), anxiety is a generalised state and is to be distinguished from fear, which is linked to a specific threat and therefore has a definite object. In contrast, anxiety is unconsciously organised and experienced internally, rather than projected externally.

The concept of OS is multi-scalar and has been applied to states (Mitzen, 2006; Parkes, 2015), minority groups (Botterill et al., 2020) and international conflicts (Rumelili, 2015), yet its application to individual migrants is largely unexplored. At the individual level, ontological insecurity manifests itself in certain emotional responses and behavioural coping mechanisms. As Mitzen (2006) argues, individual identity is formed and sustained through relationships; actors therefore seek to achieve ontological security especially by routinising their relations with significant others. Since continued agency requires the cognitive certainty that these routines provide, actors gradually get attached to these social relationships. We utilise here the concept of OS in accounting for the experiences of migrants who, we argue, were (in)secure as they became international migrants and after a while were differently able to live with ‘difference’ and cope without the security of family and the environment of the country of origin.

The CIM and OS approaches have in common the ability to also interpret emigration and return migration at the level of individuals exploring the thus-far-less-studied micro-scale factors (e.g. identity, onthological uncertainty and certainty) of emigration and return. Previous studies applying the CIM concept aimed to measure the returnees’ identity change and classified them into distinct groups (Lados & Hegedűs, 2016; Sussman, 2011). The concept of OS has thus far been rarely used for returnees although, like the CIM, with the help of OS it is also possible to measure the impact of individual factors in addition to structural and macro-scale factors (Botterill et al., 2020).

In this paper, we analyse the behaviour of migrants in the home–host–home country tripartite while moving, paying special attention to the role of family. Regarding the first stage, in connection with the CIM, cultural identity is established in the home country but in differing ways at the individual level, due to personal differences (e.g. marital status, educational attainment, occupation). For families, their children may be more important (e.g. ensuring a better life for them through emigration). Furthermore, some emigrants are more, and others are less attached to the culture of their home country. The different cultural conditions of the home country, therefore, establish the cultural identity of emigrants in diverse ways. From the point of view of ontological insecurity (Mitzen, 2006), individuals are satisfied or dissatisfied, for example, in terms of material well-being, opportunities for advancement in the workplace or, for example, ethnic–religious equality. We understand negative factors such as dissatisfaction and uncertainty at the individual level as onthological insecurity, which is a triggering factor and motivation for moving abroad in the hope of better onthological certainty and a better quality of life.

The second geographical station for emigrants is staying abroad. Here, the identity of migrants is shaped by experiences collected in the host country. As a result of the combination of home country identity and experiences acquired abroad, the identity of migrants changes, according to the four distinct identity shifts described by Sussman (2011). At the same time, the concept of OS can also be applied in terms of how much individuals’ onthological insecurity changes when leaving the home country and settling in the host country. This is linked to Cassarino’s theory of preparedness and resource mobilisation, according to which the success of individual emigration and return depends on how much and what quality of information is collected previously about the foreign destination to which individuals intend to emigrate and, in this connection, on their ability to mobilise their resources (Cassarino, 2004). If this collection of information is successful, the individual thus makes a better-informed decision (and also the return decision afterwards). This reduces onthological insecurity during the stay abroad and promotes better integration with the culture of the host society. For this reason, individuals who prepare well for their decision are more likely to belong to Sussman’s additive and global-identity groups (Sussman, 2011). In the case of families, the situation of children is of particular importance. Even if their parents are more satisfied with foreign conditions and their identity is strong, they consider the gradual loss of their child’s mother tongue and their cultural assimilation into the receiving environment to be a serious loss. This suggests that identity changes may affect family members differently, resulting in conflicts triggered by onthological insecurity.

The return, which is the third geographical phase of migration in our model, is once again a challenge for migrants who behave and react differently in the light of their experiences acquired in the home country and abroad. According to the CIM, returning to the home country is generally less of a shock for people with global identities and additives who have been well-integrated abroad. They maintained and developed their personal networks both at home and abroad; therefore, return does not affect their identity as much as it does others. Return migration for the other groups can cause more difficulties. From an OS perspective, the difficulties of returning are interpreted as follows. The level of insecurity felt in the home country and abroad determines the degree of onthological insecurity when returning home. For example, if a returnee maintains strong relationships with people both at home and in the host country, the degree of insecurity remains lower during and after the return. At the same time, maintaining strong links with the host society abroad will also make it easier for returnees to emigrate again because, for example, such individuals have more information about conditions in the foreign country than others which, in turn, once again allows these people to reassess their chances abroad in advance and prepare for possible re-emigration (Cassarino, 2004; Sardinha & Cairns, 2017).

## Research Methods

Return migrants are defined in different ways (Cassarino, 2004; King, 2000; OECD, 2008; van Houte & Davids, 2008). In this research, we considered return migrants as those who are economically active (aged over 15 years) and have ‘voluntarily’ returned to Hungary after being international migrants (i.e. working and living abroad) for at least 1 year.

A total of 48 in-depth interviews were carried out with migrants who returned to Hungary between 2012 and 2015 after a stay abroad of between 1 and 10 years. As Dustmann (2001) has argued, the length of stay abroad ideally has to be optimised: it should be long enough to allow migrants to acquire (professional) skills which might be profitable after their return but, according to King (1986), it should not be too long either, because return migrants may become disconnected and alienated from the home society. The snowball sampling technique was used for selecting interviewees. The shortcomings of this method are demonstrated by Babbie (2010: 193), who argues that the technique is not representative and should be ‘used primarily for exploratory purposes’. Since we asked our respondents to locate further interviewees whom they knew, the narratives and characteristics of interviewees might be similar. However, we deliberately tried to reach a healthy mix during the sampling (by age, sex, occupation, etc.) in order to avoid repetition. The interviews took between 50 and 90 min. The texts were transcribed and the analysis was made by NVivo software. The names of the interviewees are fictional.

Our sample is fairly similar to other international and national research in this field (e.g. Horváth, 2016; Martin & Radu, 2012). The majority of the sample was comprised of men aged 20–34 years with tertiary education. A slight majority (58%) of respondents occupied highly qualified jobs (professionals, research personnel, etc.) and some had PhDs. In our sample, men were over-represented (36 out of 48), especially among highly skilled migrants (22 out of 28). There was a slight difference between the share of singles and married returnees (23 vs 25) but singles were over-represented among low-skilled migrants. Regarding one of the key factors of this study—the family—only one-third of the interviewees returned with children.

The majority (77%) of interviewees lived in a single country before returning. The UK and the USA were over-represented among the target countries, while Germany and Austria were under-represented compared to the official statistics. However, the size of the sample offered important insights into the returnees’ complex experiences.

Interviewees were divided into two groups—high- and low-skilled returnees; this classification took place according to their foreign work experience and not their level of education. Those who worked in hospitality, construction and the food industry or had other manual jobs abroad (ISCO 3–9) were considered low-skilled migrants. Those who worked as researchers, IT professionals, engineers or medical doctors (ISCO 1–2) were identified as highly skilled.

Most of the low-skilled were only partially integrated into the host society—partly due to their weak language ability—and therefore, their cultural identity and emotional attachment to their homeland remained relatively strong. Highly skilled returnees were more likely to have migrated over greater geographical distances to achieve their goals and were also more likely to live in two or more countries. Migration paths also differed by marital status—those who emigrated with their family changed their location less frequently.

## The Spatial Dimensions of East–West Migration in Europe

Analysing migration flows within Europe spatially, one body of literature emphasises the relevance of core–periphery relations whereby ever-growing flows of labour from the periphery are oriented towards high-income countries within Europe (Martin & Radu, 2012). Countries of origin of this type of labour migration may also be Southern European (e.g. Spain, Portugal or Greece) but the weight of CEE countries in intra-EU migration has clearly increased over the last two decades. Even some of the previous peripheries of the EU (e.g. Ireland, Spain, Finland) have become destinations for immigrants from the new member states (Kahanec & Zimmermann, 2011).

The number of emigrants per 10,000 citizens is the highest in Romania, Lithuania, Croatia and Bulgaria and the lowest in Czechia, Slovenia and Hungary, which are the economically more-advanced and politically more-stable CEE countries (Table 1). In absolute terms, the number of emigrants is highest from the more populous countries such as Romania (3.5 million) and Poland (2.5 million). These two countries represent two-thirds of CEE migrants in the EU. With around 450,000 emigrants, Hungary ranks fifth among post-communist countries in terms of absolute numbers.

**Table 1:** Central and Eastern European emigrants living in other countries of the European Union per 10,000 inhabitants (2004–2019)

Country	2004–2007	2008–2011	2012–2015	2016–2019
Romania	389	860	1199	1672
Lithuania	172	500	819	1233
Croatia	765	564	759	1129
Bulgaria	231	375	596	1112
Latvia	91	195	653	958
Poland	158	313	472	642
Estonia	189	281	437	636
Slovakia	180	252	407	623
Hungary	83	105	256	439
Slovenia	162	127	197	306
Czechia	55	61	81	151
**East–Central Europe**	**218**	**385**	**576**	**835**

*Source:* Authors’ calculations based on the Eurostat database (see ‘Population on 1 January by five-year age group, sex and citizenship’)

East–West migration involves the skilled and mobile sections of the originating societies. According to Blaskó & Gödri (2016), the majority of Hungarian emigrants are young males, generally aged 20–39 years. Furthermore, the share of people with higher education and those who are unmarried is greater among emigrants than the country’s average. Recent research confirms that migration intentions are the highest among young people, particularly the 21–30 age group (Siskáné Szilasi & Halász, 2018).

This positionality against the broader geographies of intra-EU migration puts the Hungarian migrants in a particular state of ontological (in)security. With greater mobility freedom as part of their EU citizenship and more-feasible options for return—not least due to a growing economy—their migration and return decisions should be less constrained when compared to migrants from other parts of the world.

However, barriers can still hinder their migratory experiences. In the case of Hungary, language is a strong barrier in every situation; nevertheless, historical connections and geopolitical orientation result in the dominance of Germany as a receiving country (44%)—especially the southern federal states of Bavaria and Baden-Württemberg—and Austria (19%). In addition to historical links, the role of global cities (e.g. London, Paris, Amsterdam, Brussels, Milan) is also detectable in Hungarian migration, especially among the younger and highly skilled migrants, who belong mostly to the creative and professional classes.

The proportion of people returning home to CEE has also increased over time (Table 2). The reasons are manifold. First, many people had to return after the global financial crisis of 2008 and subsequent years, as labour-market opportunities shrank in the West. Secondly, higher economic growth rates in CEE countries compared to EU-15 countries resulted in a slow but gradual levelling out of wages after EU accession, which significantly influenced individual decisions regarding emigration and return migration. Thirdly, several programmes and initiatives supporting return migration and tackling brain drain were developed and implemented by CEE countries at various geographical scales (i.e. national, regional or local level) which started to bear fruit in the 2010s (Kovács et al., 2013).

**Table 2:** The proportion of returnees in Central and Eastern Europe (2004–2019)

	Proportion of returnees compared to immigrants (all data %)				Number of returnees per 10,000 inhabitants			
	2004–2007	2008–2011	2012–2015	2016–2019	2004–2007	2008–2011	2012–2015	2016–2019
Romania	No data	92.1	90.5	82.4	No data	64.6	66.8	72.6
Estonia	39.1	50.0	56.0	45.1	6.1	13.2	27.7	58.3
Lithuania	68.8	79.9	84.1	56.0	14.9	23.3	62.7	54.1
Hungary	8.1	9.7	49.7	43.8	2.1	2.9	23.3	32.8
Poland	87.0	68.1	54.6	54.6	2.5	23.1	31.5	30.8
Latvia	No data	32.5	60.9	46.1	No data	8.6	31.3	23.9
Bulgaria	96.0	No data	35.3	54.3	0.5	No data	10.3	21.9
Croatia	92.8	60.7	49.4	36.6	25.8	16.7	12.1	20.7
Slovenia	9.1	12.8	17.7	15.0	8.5	14.2	12.5	17.2
Slovakia	No data	18.8	49.4	58.3	No data	2.2	5.2	7.8
Czechia	2.9	28.3	18.0	6.2	2.0	17.6	5.3	4.2
**East–Central Europe**	**20.3**	**61.8**	**61.4**	**53.1**	**3.1**	**25.1**	**31.7**	**34.6**

*Source:* Own calculations based on Eurostat database (see ‘Immigration by age group, sex and citizenship’; ‘Population on 1 January by age and sex’)

In parallel to the East–West migratory wave, the number of returning Hungarians has also continuously increased. To date, only a few studies have been conducted to analyse the spatial characteristics of return within the home country (Apsite-Berina et al., 2018; Kincses, 2015; Vathi et al., 2019). According to the 2011 national and 2016 micro censuses, returning Hungarians adopt certain attitudes when choosing their place of residence after return, settling mainly in the metropolitan region of Budapest, in the vicinity of Lake Balaton and in more populous regional centres. According to Kincses (2015), only 31% of homebound migrants returned to their previous residence.

A complex and comprehensive migration strategy has not yet been developed in Hungary; however, some initiatives focusing on specific target groups or areas of migration have been implemented. One of the most well-known and successful initiatives is the *Lendület* (‘Momentum’) programme founded by the Hungarian Academy of Sciences in 2009, which aims to attract back young Hungarian researchers who have left the country. Another initiative is an NGO called ‘*Gyere Haza Alapítvány*’ (‘Come Back Home Association’), founded in 2010. In 2013, it expanded its services to offer online and personal support to those emigrants wishing to return. We can identify several retain-type programmes as well—for example, in the healthcare system or the Act CCIV of 2011 on National Higher Education. In 2015, the so-called *Gyere Haza* (‘Come Back Home’) programme was launched to re-attract young, highly educated Hungarians living in London and offered re-employment after return; the programme ended in 2016 (Lados & Hegedűs, 2016). Despite similar initiatives in other CEE countries, no comprehensive migration policy dealing with out- and return migration in the European Union exists to date (Boros & Hegedűs, 2016; Kálmán, 2016; Nadler et al., 2016).

## Empirical Research Findings

### Moving Abroad

Our research investigated the migratory behaviour, working conditions and family background of Hungarian return migrants, focusing on the motivations and circumstances of both emigration and return migration among high- and low-skilled migrants. In synthesis, we find that emigration was indeed a search for ontological security, even though this was better carried out by the highly skilled.

The vast majority of the interviewees left Hungary with definite plans. *Emigration* was expected to be a positive catalyst in their life, anticipating either tangible or intangible assets. Regarding the motivations for emigration, high- and less-skilled people differed considerably. According to the literature, the most important motivations for the emigration of CEE people were the higher standard of living and income expected abroad, professional advancement and the desire for adventure (Barcevicius et al., 2012; Lang & Nadler, 2014). Of these, not surprisingly, the highly educated were motivated mostly by professional advancement (gaining new skills, experience, learning new cultures and building networks) whereas for the less-skilled, higher (and more stable) incomes, adventure and the insecurity of family in the home country were the primary considerations of emigration.

More-conscious preparation for emigration was typical among highly qualified people whose foreign jobs matched their qualifications. Most considered emigration as a transitional period in their life, as described by the preparedness theory (Cassarino, 2004), the most important aspect for them being the achievement of professional success and—sometimes pre-planned—future career-building which, in some professions, has ‘an untold, but predictable, almost mandatory step’ (Tamas, 39 male, researcher). For them, although the stay abroad itself was important, the geographical location also played a significant role:

Of course, in the beginning, it sounded very interesting and exciting that I will live in Australia but, after some years, it became too exhausting to travel across the whole world to participate in a conference, visit my relatives or see anything different. There [in Australia] you can travel hundreds of kilometres but nature is not changing. Here [in Europe] if you travel hundreds of kilometres you can enjoy various natural and cultural environments. It is priceless (Peter, 37, male, researcher).

By contrast, not everyone in the low-skilled group of migrants was consciously prepared to work abroad; usually emigration was the result of a quick decision and many of them did not even speak about the host country language. The move could be regarded as a kind of forced survival strategy rather than a conscious career-building process (Siskáné Szilasi & Halász, 2018), underpinned by a strong sense of ontological security. As expressed by Eniko (30, female, trained worker):

Our goal was to save enough money abroad (…) to have a chance to buy an apartment later when we come back [to Hungary] or start our own business (…) We never planned to stay forever and integrate into the foreign society.

Next to financial security and the survival of the family, capital accumulation was also more often mentioned as a rationale for migration by lower-skilled migrants (Cingolani & Vietti, 2020). As Zoltan (39, male, butcher) says:

They [family members] came out, had a look around, checked the living conditions, everything. Then we decided that they also follow me. Why should I pay the expenses of two households? One at home and one abroad. The house at home remained empty, we did not even heat it during winter. We did not bother about our house back home.

Exclusionary attitudes of the public and discriminating legislation in the home country also contributed to emigration, in some cases among minorities where ontological insecurity was indeed a key push factor for leaving Hungary. Decisions on emigration were difficult in these cases, taking into consideration factors such as family, career or identity; however, as Szabolcs (30, male, translator) posited, possible future discrimination influencing everyday life and the vision of an exclusionary society encouraged emigration:

My first thoughts about leaving the country came when the constitution was modified in December 2012, if I remember well. You know I am a homosexual. After a while I became anxious what the amendment of the constitution would bring, how it would affect gays. My anxiety has not decreased since then. I can expect that, in the future, the situation will change in this country in that direction, so that I will need to leave forever. I play a very visible role in the local gay community and I do not know how it will impact on me and my partner. We have started to think of leaving the country for good.

Thus, moving abroad was driven by different factors in the groups of low- and high-skilled migrants. Seeking professional advancement and career chances dominated the decision of the highly skilled, whereas better labour-market conditions, higher wages and escaping hardship in the home country prevailed among the lower-skilled. Nevertheless, there was a sense in both groups that there was a personal benefit to be gained from emigration. In carrying out their moves, the geographical distance of the host country was more relevant for lower-skilled migrants. Regular visits to the home country and maintaining strong connections with family members and friends remaining at home both played an important role as part of ontological security. Highly skilled migrants were less constrained by this aspect as, due to their professional and language skills, they were also able to plan longer-term and integrate into the host society.

### Motivations to Return

For a better understanding of the motivations of *return migration*, we considered Sussman’s (2011) CIM. Even though the number of returnees with an *affirmative* identity shift was the smallest in our sample, we were able to identify them. We also suppose that, given the volume of East–West migration, they probably have a much higher share among migrants. They were generally employed in the unskilled manual-labour sector, could barely speak any foreign language and amassed many negative experiences during their migration career (e.g. bad working conditions, cultural isolation, hostility in the host country). For them, migration was a failure (García-Pereiro, 2019); hence, return and reunion with their family were perceived positively (cf. Lulle et al., 2019).

Members of the *subtractive* group were mainly lower-skilled people who moved abroad with their families. They experienced difficulties integrating into the host society—which can be linked mainly to their lack of sufficient language ability and poor working conditions. Knowledge of a foreign language was typically not a primary requirement for those who worked in trained jobs, which means they worked with other Hungarians or immigrants from other countries who did not speak the relevant foreign language. The main motivation for them was to earn a higher salary and make financial savings (Lang & Nadler, 2014; Martin & Radu, 2012). Their social relations abroad were superficial and fragile and easily broke up after return. Their integration in the host society was also often hampered by homesickness, as Zoltan (39, male, butcher) mentioned:

I was pulled back by the family by a hundred hands. [He used to work abroad in different countries but the most difficult for him was to live separately from the rest of family.] In Ireland I felt already OK [with the family]; maybe the work was also better. But Germany was much harder, I could have cried every day, I commuted monthly.

Uncertainty and anxiety as factors of return were also palpable among subtractive returnees. Return intentions were prompted by negative experiences and personal goals, including family formation or buying their own apartment (de Jong & de Valk, 2020):

I saw how much we worked abroad [together with my Hungarian friends] and we did not live very much better than those who stayed at home. I saw my school mates with kids who bought houses and cars and regularly went to Greece for a holiday. And we worked like fools. At that point I realised that we were not at all in the right place (Ferenc, 30, male, unskilled worker).

The group of *additive* returnees consisted of highly skilled migrants who moved with their families on the one hand and, on the other, those who took jobs below their qualifications, usually routine work (i.e. brain waste). Family ties were very important motivations to return. In addition, the fear that their children would completely assimilate abroad and would lose their Hungarian identity motivated many interviewees to return home. In these cases, the sense of ontological insecurity concerned their parenting role and the cohesiveness of their families, as Gergo (32, male, translator) posited:

At home we spoke only Hungarian. We had ambivalent feelings, we were not anxious, we only regretted that it was over. But we felt it [return] had to happen and it will be better in the long-term, especially for the kids. We did not want to stay abroad any longer because we wanted the kids to remain Hungarians. If we had stayed they would have become Luxembourger or European, or nobody.

Anxiety about assimilation and losing their roots in the homeland reaffirmed migrants’ Hungarian identity. Family connections (especially elderly parent care) also influenced the decision on return migration (Bryceson, 2019), as Marta (33, female, hotel receptionist) illustrated:

A bit, I felt that my parents are old, they brought me up, got me educated and I simply escape because I have difficulties and cannot subsist myself. Others can make a living of sorts and don’t escape. They [the parents] are getting older, and one day they may need assistance and how can I help them from two thousand kilometres? ‘Don’t give up! Speedy recovery!’ I am not in England now, only in Budapest but, from here, I can get home within two and a half hours if anything happens.

According to the CIM, *global identity* returnees are those who change the most during their stay in the host country. In our sample, they were typically highly skilled people with or without a family. For them, one of the most important changes in their life was the search for a new identity (Guo, 2016; Tedeschi et al., 2020). As Szabolcs (30, male, translator) expressed it, ‘I am first of all European, secondly a Budapest guy and thirdly Hungarian’. For them, reintegration into the labour market at home was very easy due to the skills and experiences acquired abroad (see also Konzett-Smoliner, 2016). The emotional aspect of reintegration, however, was a challenge for some, as they were also strongly attached to the host country’s culture (habits, practices, locations, etc.) even when returning, despite some instances of discrimination in the workplace (Remennick, 2021). As Nandor (32, male, IT professional) argued, he even had ‘homesick’ feelings towards the host country after his return:

When I came home from London I wanted to be back there for several months. I had to keep control over myself not to desire to go back so much. It took a long time. When I was already at home I had to go back to London three times because of the work. On every occasion when I went back I started to cry in the first one or two hours. I am not a sentimental type, but I could not hold on. Simply, when I arrived at Liverpool Station by train from the airport, and I saw the crowd, everybody was running to work, I simply started to cry. I was so much attached to London emotionally. And I did not feel this in Budapest, although I like Budapest.

Another global-identity returnee also referred to emotional difficulties after return, which is argued by the literature both in the EU (Sardinha & Cairns, 2017) and in the wider intra-European context (Vathi et al., 2019). As Gabor (29, male, teacher) stated, during his stay abroad he got accustomed to the local circumstances so much that it was a real challenge for him to reintegrate into everyday life in Hungary:

It was very difficult to reintegrate. What is annoying in Hungary is the mentality. The attitude of people here, their mentality and behaviour could make me so disappointed that I turned inside myself for days.

To sum up, the motivations for return were very variable among returnees. The dissimilarity in the lifestyles of the home and host countries, migrants’ inability to integrate in the host society, their pursuit of personal goals and a ‘sense of nostalgia’ towards the home country all played a role in people’s decision to return, although at different rates in the various identity groups. However, we can clearly see that family relations and friendship ties to the homeland were among the primary reasons for return, over and above economic and social considerations in most cases. It also became clear that returnees’ expectations that return would increase their psychosocial well-being often did not match the post-return reality.

## Discussion and Conclusions

We have analysed the motivations and identity changes of Hungarian emigrants who returned to their home country after an extended stay in the West. Our findings confirmed that migration and return are complex, multi-dimensional processes influenced by many factors and underpinned to differing extents by a sense of ontological (in)security. The role of family as a geopolitical unit is different in the four identity groups (affirmative, subtractive, additive and global-identity shifters—Sussman, 2011). Factors like efforts to hold the family together, fears over children’s assimilation, loss of identity or uncertainty abroad are the most important for subtractive and additive returnees (Bhugra & Becker, 2005; Sinatti & Horst, 2014). Among affirmative identity-shift returnees, family also plays a role and their attachment to the home culture and family remains strong while living abroad. Negative experiences in the host country (cultural isolation, marginalisation in the workplace, etc.) have highlighted the role of the family, which ultimately often results in return migration. Out of the four groups of the CIM (Sussman, 2011), global-identity returnees are the least affected by the family as a geopolitical unit. They are the most successful, most mobile migrants who can easily shift between identities and adapt to new circumstances quickly and are the least dependent on family relations. Instead, they appear to experience ontological insecurity in conditions of immobility and a lack of cosmopolitanism.

Our results also showed that factors influencing return migration differ considerably between the high- and the low-skilled in general. High-skilled returnees are more mobile; they can integrate better into the host society and are able to benefit from their widening social and professional networks after return (Konzett-Smoliner, 2016). Low-skilled returnees normally consider their stay abroad as temporary. Their main goal is to earn a reasonable amount of money within a couple of years; they barely integrate into the host society and many of them consider the stay abroad as a failure after return. Their ontological security is based on factors which are differently contingent upon migration and return.

As emigration from CEE countries accelerated, some of them started to draw up and implement return-migration initiatives in order to halt the process of brain drain and make brain gain possible. Common in these return programmes is an emotional emphasis on the natural beauties and cultural traditions (e.g. art, gastronomy) of the home country and its recent dynamic economic development and improved living standards. However, we can also find nationalistic and anti-Western rhetoric in some of these programmes. The Hungarian ‘Wayback’ programme lists negative features of Western (receiving) countries as opposed to the homeland (e.g. higher income tax, more expensive health services, business start-up difficulties) among the possible reasons to return. The narrative therefore appears to target experiences of ontological (in)security among migrants.

For a smoother and more-balanced migration and return migration between East and West, institutions at the highest European level should be addressed (i.e. the European Parliament and European Commission). Policies and programmes that tackle the challenges would be needed, utilising the lessons of previous supranational and national strategies and programmes in the world (Ho, 2013; Nadler et al., 2016). Such initiatives and programmes should consider the differences in the motivations and behaviour of the various groups of returning migrants. Using the CIM and OS concepts, this study provides empirical results for potential groups of returnees and their characteristics. Moreover, as some EU-financed projects have previously highlighted (Lang & Nadler, 2014; Smoliner et al., 2013; Soltesz, 2019), the reattraction, re-employment and reintegration of migrants should equally play a key role in these initiatives. We do not yet know the combined effects of Brexit and the COVID-19 pandemic on East–West migration but, most probably, the willingness of ‘Easterners’ to migrate to the West will decrease in the future, unless new, exceptional circumstances arise, such as the currently ongoing Russian invasion of Ukraine. Nevertheless, the challenges of return migration and reintegration will remain. Our paper has offered evidence on the key factors that motivate migration and return by highlighting the role of the socio-economic background and identification patterns in providing a sense of ontological (in)security which emerges as a driver of these motivations.
